# A checklist of spiders in tea plantations of China

**DOI:** 10.3897/BDJ.8.e60143

**Published:** 2020-12-08

**Authors:** Xuhao Song, Tingbang Yang, Xiaoqin Xu, Yang Zhong

**Affiliations:** 1 Key Laboratory of Southwest China Wildlife Resources Conservation (Ministry of Education), China West Normal University, Nanchong 637009, Sichuan, China Key Laboratory of Southwest China Wildlife Resources Conservation (Ministry of Education), China West Normal University Nanchong 637009, Sichuan China; 2 Institute of Ecology, China West Normal University, Nanchong 637009, Sichuan, China Institute of Ecology, China West Normal University Nanchong 637009, Sichuan China; 3 Hubei Key Laboratory of Radiation Chemistry and Functional Materials, School of Nuclear Technology and Chemistry & Biology, Hubei University of Science and Technology, Xianning 437100, Hubei, China Hubei Key Laboratory of Radiation Chemistry and Functional Materials, School of Nuclear Technology and Chemistry & Biology, Hubei University of Science and Technology Xianning 437100, Hubei China

**Keywords:** tea plantations, spider, checklist, biodiversity, China

## Abstract

**Background:**

Spiders are the most dominant predatory natural enemies of insect pests in the tea plantation ecosystem. There has been a large amount of literature published about the investigation of spider species in Chinese tea plantations from 1982 to 2020. Here, the spider species in Chinese tea plantations has been summarised and the dominant spider species in each regional tea plantation recorded. To date, there were 535 spider species from 40 families reported in Chinese tea plantations.

**New information:**

There are 245 spider species from 13 families now being added to the checklist. A total of 89 spider species from 19 families were the dominant species, amongst them, *Agelena labyrinthica*, *Allagelena difficilis*, *Neoscona theisi*, *Clubiona deletrix*, *Clubiona japonicola*, *Hylyphantes graminicola*, *Pardosa laura*, *Oxyopes sertatus*, *Evarcha albaria*, *Plexippus paykulli*, *Coleosoma octomaculatum*, *Ebrechtella tricuspidata* and *Xysticus ephippiatus* were recorded in many tea plantations. The checklist will provide important data for the biodiversity and distribution of spiders in tea plantations of China.

## Introduction

**Figure 1.**
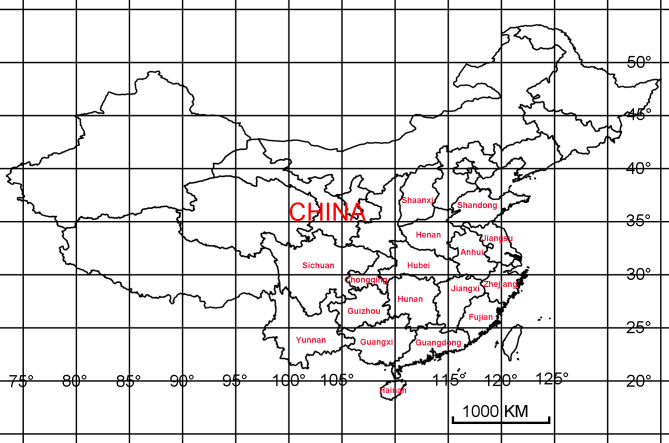
The spider species are summarised from tea plantations of 16 Provinces and one Municipality of China.

Compared with other farmland ecosystems, tea plantations are relatively stable ecosystems, containing many natural enemies of pests and these ecosystems provide favourable conditions for the protection and utilisation of natural enemies for pest control (Ye et al. 2014). The main invertebrate predators of tea pests are Araneae, Coleoptera, Hemiptera, Neuroptera, Mantodea, Odonata and Diptera (Das et al. 2010). Amongst them, spiders are the most dominant predatory natural enemies in the tea plantation ecosystem and the number of their occurrence accounts for 65.0%-97.8% of predatory natural enemies (Chen et al. 2004). Chen et al. (2000) performed a comprehensive investigation on spider species in Chinese tea plantations from 1983 to 1999 and reported a total of 290 spider species from 27 families. Since then, spider species have continually been reported in Chinese tea plantations and there has been a large amount of literature published about the investigation of spider species in tea plantations. Here, literature about the investigation of spider species in Chinese tea plantations has been collated. The spider species from 16 Provinces (Anhui, Fujian, Guangdong, Guangxi, Guizhou, Hainan, Henan, Hubei, Hunan, Jiangsu, Jiangxi, Shaanxi, Shandong, Sichuan, Yunnan and Zhejiang) and one Municipality (Chongqing) of China have been summarised (Fig. 1). The geographical distribution of each spider species and the dominant spider species in each regional tea plantation have been recorded in detail. The checklist will provide important data for the biodiversity and distribution of spiders in tea plantations of China.

## Geographic coverage

### Description

A total of 16 Provinces (Anhui, Fujian, Guangdong, Guangxi, Guizhou, Hainan, Henan, Hubei, Hunan, Jiangsu, Jiangxi, Shaanxi, Shandong, Sichuan, Yunnan and Zhejiang) and one Municipality (Chongqing) of China have been investigated.

### Coordinates

 and Latitude; and Longitude.

## Taxonomic coverage

### Taxa included

**Table:** 

Rank	Scientific Name	Common Name
order	Araneae	Spiders

## Usage licence

### Usage licence

Open Data Commons Attribution License

## Data resources

### Data package title

doi_10.5061_dryad.0rxwdbrxx__v4

### Resource link

https://datadryad.org/stash/share/0lrvPzBdsJujEGPqSwK4Wl9_nUIA5b58D4gSgeBDs_4

### Alternative identifiers

https://doi.org/10.5061/dryad.0rxwdbrxx

### Number of data sets

2

### Data set 1.

#### Data set name

Checklist.txt

#### Number of columns

4

#### 

**Data set 1.** 

Column label	Column description
Family	Taxonomic level of family.
Species	Taxonomic level of species.
Distribution	Geographical distribution of spiders in tea plantations of China.
References	Source of information on spider species.

### Data set 2.

#### Data set name

List_of_References.txt

#### Number of columns

1

#### 

**Data set 2.** 

Column label	Column description
List of references	A list of the references cited in Checklist.txt.

## Additional information

To date, there were 535 spider species from 40 families reported in Chinese tea plantations, with a total of 13 families and 245 species now being added compared with those reported by Chen et al. (2000). A total of 89 spider species from 19 families were the dominant species, amongst them, *Agelena labyrinthica*, *Allagelena difficilis*, *Neoscona theisi*, *Clubiona deletrix*, *Clubiona japonicola*, *Hylyphantes graminicola*, *Pardosa laura*, *Oxyopes sertatus*, *Evarcha albaria*, *Plexippus paykulli*, *Coleosoma octomaculatum*, *Ebrechtella tricuspidata* and *Xysticus ephippiatus* were recorded in many tea plantations (Table 1).

## Figures and Tables

**Table 1.** Dominant spider species in tea plantations of China.

**Family**	**Species**	**References**
Atypidae	*Atypus heterothecus* Zhang, 1985	Cui et al. 2012
Atypidae	*Atypus sinensis* Schenkel, 1953	Zhu et al. 2011
Agelenidae	*Agelena labyrinthica* (Clerck, 1757)	Zhang 1983, Xu et al. 1995, Han et al. 2006, Dai and Han 2009, Zhu et al. 2011
Agelenidae	*Agelena limbata* Thorell, 1897	Dai and Han 2009
Agelenidae	*Allagelena difficilis* (Fox, 1936)	Dai and Han 2009, Zhu et al. 2011, Cui et al. 2012, Li 2012, Wang et al. 2013
Agelenidae	*Pireneitega taishanensis* (Wang, Yin, Peng & Xie, 1990)	Li et al. 2012
Araneidae	*Acusilas coccineus* Simon, 1895	Li et al. 2012
Araneidae	*Araneus diadematus* Clerck, 1757	Xu et al. 1995
Araneidae	*Araneus ejusmodi* Bösenberg & Strand, 1906	Li et al. 2012
Araneidae	*Argiope amoena* L. Koch, 1878	Dai and Han 2009, Li et al. 2012
Araneidae	*Argiope minuta* Karsch, 1879	Li et al. 2012
Araneidae	*Cyclosa atrata* Bösenberg & Strand, 1906	Li et al. 2012
Araneidae	*Cyclosa octotuberculata* Karsch, 1879	Li et al. 2012
Araneidae	*Cyclosa sedeculata* Karsch, 1879	Li et al. 2012
Araneidae	*Eriovixia laglaizei* (Simon, 1877)	Li et al. 2012
Araneidae	*Hypsosinga sanguinea* (C. L. Koch, 1844)	Dai and Han 2009
Araneidae	*Lariniaria argiopiformis* (Bösenberg & Strand, 1906)	Dai and Han 2009
Araneidae	*Larinioides cornutus* (Clerck, 1757)	Dai and Han 2009
Araneidae	*Neoscona adianta* (Walckenaer, 1802)	Zhang 1983, Dai and Han 2009, Li et al. 2012
Araneidae	*Neoscona punctigera* (Doleschall, 1857)	Zhu et al. 2011
Araneidae	*Neoscona scylla* (Karsch, 1879)	Dai and Han 2009
Araneidae	*Neoscona scylloides* (Bösenberg & Strand, 1906)	Li et al. 2012
Araneidae	*Neoscona theisi* (Walckenaer, 1841)	Zhang 1983, Liu et al. 1994, Li et al. 2012, Liu et al. 2015
Araneidae	*Nephila clavata* L. Koch, 1878	Dai and Han 2009
Araneidae	*Nephila pilipes* (Fabricius, 1793)	Dai and Han 2009
Araneidae	*Singa hamata* (Clerck, 1757)	Han et al. 2006, Li et al. 2012
Clubionidae	*Clubiona corrugata* Bösenberg & Strand, 1906	Zhang et al. 1996, Chen 2003
Clubionidae	*Clubiona deletrix* O. Pickard-Cambridge, 1885	Zhang et al. 1996, Chen 2003, Dai and Han 2009, Xiong et al. 2010, Li et al. 2012, Liu et al. 2015
Clubionidae	*Clubiona duoconcava* Zhang & Hu, 1991	Cui et al. 2012
Clubionidae	*Clubiona japonicola* Bösenberg & Strand, 1906	Xie 1995, Dai and Han 2009, Xiong et al. 2010, Chen 2016
Ctenidae	*Anahita fauna* Karsch, 1879	Cui et al. 2012, Li et al. 2012
Gnaphosidae	*Zelotes asiaticus* (Bösenberg & Strand, 1906)	Dai and Han 2009, Huang et al. 2012, Li 2012, Li et al. 2012, Gao 2014
Hahniidae	*Hahnia zhejiangensis* Song & Zheng, 1982	Gao 2014
Linyphiidae	*Gnathonarium gibberum* Oi, 1960	Dai and Han 2009
Linyphiidae	*Hylyphantes graminicola* (Sundevall, 1830)	Hou and Zhao 1982, Zhang 1983, Xie 1995, Xu et al. 1995, Han et al. 2006, Zeng et al. 2008, Dai and Han 2009, Feng et al. 2010, Li et al. 2010, Li et al. 2012, Feng et al. 2013, Wang et al. 2013, Liu et al. 2015
Linyphiidae	*Neriene cavaleriei* (Schenkel, 1963)	Cui et al. 2012, Li et al. 2012
Linyphiidae	*Neriene oidedicata* van Helsdingen, 1969	Chen 1992
Linyphiidae	*Neriene radiata* (Walckenaer, 1841)	Zhang 1983, Xu et al. 1995
Linyphiidae	*Ummeliata insecticeps* (Bösenberg & Strand, 1906)	Dai and Han 2009
Lycosidae	*Lycosa coelestis* L. Koch, 1878	Huang et al. 2012, Li 2012, Li et al. 2012
Lycosidae	*Pardosa astrigera* L. Koch, 1878	Dai and Han 2009, Li et al. 2012, Li et al. 2012
Lycosidae	*Pardosa laura* Karsch, 1879	Dai and Han 2009, Cui et al. 2012, Li 2012, Li et al. 2012, Wang et al. 2013, Xing and Zhu 2015, Xing et al. 2016
Lycosidae	*Pardosa pseudoannulata* (Bösenberg & Strand, 1906)	Xing and Zhu 2015, Xing et al. 2016
Lycosidae	*Pardosa tschekiangiensis* Schenkel, 1963	Dai and Han 2009
Lycosidae	*Pirata subpiraticus* (Bösenberg & Strand, 1906)	Xing and Zhu 2015, Xing et al. 2016
Lycosidae	*Piratula procurva* (Bösenberg & Strand, 1906)	Huang et al. 2012
Lycosidae	*Trochosa ruricoloides* Schenkel, 1963	Dai and Han 2009
Lycosidae	*Trochosa suiningensis* Peng, Yin, Zhang & Kim, 1997	Li et al. 2012
Miturgidae	*Prochora praticola* (Bösenberg & Strand, 1906)	Li et al. 2012, Gao 2014
Oxyopidae	*Oxyopes fujianicus* Song & Zhu, 1993	Li et al. 2012
Oxyopidae	*Oxyopes hotingchiehi* Schenkel, 1963	Li et al. 2012
Oxyopidae	*Oxyopes macilentus* L. Koch, 1878	Li et al. 2012
Oxyopidae	*Oxyopes sertatus* L. Koch, 1878	Zhang 1983, Liu et al. 1994, Zhang et al. 1996, Chen 2003, Han et al. 2006, Zeng et al. 2008, Dai and Han 2009, Feng et al. 2010, Deng and Tan 2002, Li et al. 2010
Philodromidae	*Philodromus rufus* Walckenaer, 1826	Dai and Han 2009
Pholcidae	*Pholcus crypticolens* Bösenberg & Strand, 1906	Li et al. 2012
Pisauridae	*Dolomedes saganus* Bösenberg & Strand, 1906	Dai and Han 2009
Salticidae	*Bianor angulosus* (Karsch, 1879)	Bai et al. 2011, Li et al. 2012
Salticidae	*Evarcha albaria* (L. Koch, 1878)	Liu et al. 1994, Xie 1995, Zhang et al. 1996, Chen 2003, Han et al. 2006, Zhu et al. 2011, Cui et al. 2012, Li 2012, Li et al. 2012, Li et al. 2010, Gao 2014
Salticidae	*Hasarius adansoni* (Audouin, 1826)	Dai and Han 2009, Li et al. 2012
Salticidae	*Mendoza canestrinii* (Ninni, 1868)	Dai and Han 2009
Salticidae	*Myrmarachne gisti* Fox, 1937	Li et al. 2012
Salticidae	*Myrmarachne inermichelis* Bösenberg & Strand, 1906	Li et al. 2010
Salticidae	*Phintella arenicolor* (Grube, 1861)	Zhang et al. 1996, Chen 2003, Dai and Han 2009
Salticidae	*Phintella bifurcilinea* (Bösenberg & Strand, 1906)	Chen 1992
Salticidae	*Phintella versicolor* (C. L. Koch, 1846)	Xie 1995, Li et al. 2010, Li et al. 2012
Salticidae	*Plexippus paykulli* (Audouin, 1826)	Liu et al. 1994, Han et al. 2006, Dai and Han 2009, Li et al. 2012
Salticidae	*Plexippus setipes* Karsch, 1879	Xie 1995, Li et al. 2010
Salticidae	*Sibianor aurocinctus* (Ohlert, 1865)	Dai and Han 2009, Bai et al. 2011
Salticidae	*Siler cupreus* Simon, 1889	Dai and Han 2009
Tetragnathidae	*Leucauge blanda* (L. Koch, 1878)	Xing and Zhu 2015, Xing et al. 2016
Tetragnathidae	*Pachygnatha quadrimaculata* (Bösenberg & Strand, 1906)	Li et al. 2012
Tetragnathidae	*Tetragnatha maxillosa* Thorell, 1895	Liu et al. 1994, Dai and Han 2009, Liu et al. 2015
Tetragnathidae	*Tetragnatha praedonia* L. Koch, 1878	Chen 1992, Dai and Han 2009, Chen 2016
Tetragnathidae	*Tetragnatha squamata* Karsch, 1879	Xu et al. 1995, Xiong et al. 2010
Theridiidae	*Chrosiothes sudabides* (Bösenberg & Strand, 1906)	Li et al. 2012
Theridiidae	*Chrysso argyrodiformis* (Yaginuma, 1952)	Liu et al. 1994
Theridiidae	*Coleosoma blandum* O. Pickard-Cambridge, 1882	Xing and Zhu 2015, Chen 2016, Xing et al. 2016
Theridiidae	*Coleosoma octomaculatum* (Bösenberg & Strand, 1906)	Hou and Zhao 1982, Zhang 1983, Liu et al. 1994, Xie 1995, Xu et al. 1995, Zhang et al. 1996, Chen 2003, Han et al. 2006, Zeng et al. 2008, Dai and Han 2009, Li et al. 2010, Li 2012, Li et al. 2012, Feng et al. 2013, Liu et al. 2015, Chen 2016
Theridiidae	*Enoplognatha caricis* (Fickert, 1876)	Liu et al. 2015
Theridiidae	*Episinus variacorneus* Chen, Peng & Zhao, 1992	Liu et al. 1994
Theridiidae	*Meotipa pulcherrima* (Mello-Leitão, 1917)	Cui et al. 2012
Theridiidae	*Nihonhimea japonica* (Bösenberg & Strand, 1906)	Dai and Han 2009
Theridiidae	*Paidiscura subpallens* (Bösenberg & Strand, 1906)	Liu et al. 1994
Theridiidae	*Parasteatoda tepidariorum* (C. L. Koch, 1841)	Dai and Han 2009
Thomisidae	*Ebrechtella tricuspidata* (Fabricius, 1775)	Zhang 1983, Zhang et al. 1996, Chen 2003, Han et al. 2006, Zeng et al. 2008, Dai and Han 2009, Feng et al. 2010, Li et al. 2010, Zhu et al. 2011, Liu et al. 2015
Thomisidae	*Runcinia insect* (L. Koch, 1875)	Li et al. 2012
Thomisidae	*Xysticus croceus* Fox, 1937	Cui et al. 2012
Thomisidae	*Xysticus ephippiatus* Simon, 1880	Zhang et al. 1996, Chen 2003, Han et al. 2006, Dai and Han 2009, Feng et al. 2010, Li 2012, Li et al. 2012, Liu et al. 2015
Trachelidae	*Trachelas japonicus* Bösenberg & Strand, 1906	Liu et al. 1994
